# Influence of Spinning Method on Shape Memory Effect of Thermoplastic Polyurethane Yarns

**DOI:** 10.3390/polym15010239

**Published:** 2023-01-03

**Authors:** Lukas Benecke, Robert Tonndorf, Chokri Cherif, Dilbar Aibibu

**Affiliations:** 1 Institute of Textile Machinery and High Performance Material Technology, Technische Universität Dresden, 01069 Dresden, Germany; 2 Centre for Tactile Internet with Human-in-the-Loop (CeTI), Technische Universität Dresden, 01062 Dresden, Germany

**Keywords:** shape memory polymer, shape memory effect, fiber, melt/wet spinning

## Abstract

Shape memory polymers are gaining increasing attention, especially in the medical field, due to their ability to recover high deformations, low activation temperatures, and relatively high actuation stress. Furthermore, shape memory polymers can be applied as fiber-based solutions for the development of smart devices used in many fields, e.g., industry 4.0, medicine, and skill learning. These kind of applications require sensors, actors, and conductive structures. Textile structures address these applications by meeting requirements such as being flexible, adaptable, and wearable. In this work, the influence of spinning methods and parameters on the effect of shape memory polymer yarns was investigated, comparing melt and wet spinning. It is shown that the spinning method can significantly influence the strain fixation and generated stress during the activation of the shape memory effect. Furthermore, for wet spinning, the draw ratio could affect the stress conversion, influencing its efficiency. Therefore, the selection of the spinning process is essential for the setting of application-specific shape-changing properties.

## 1. Introduction

Today, shape memory materials are an emerging class of materials with outstanding properties that are being investigated in ongoing research into smart materials, sensors, actuators, and especially for minimally invasive medical applications [1,2,3]. Self-tightening sutures [4,5], cardio-vascular stents [6,7], and stimulus specific drug delivery systems [8,9] are just a few examples of the research concerning shape memory materials. Buehler et al. started this research field in 1963 by presenting the shape memory effect (SME) of the nowadays well-known nickel–titanium alloy Nitinol [10,11]. The SME describes a repeatable process, in which a shape deformation is recovered when the material is stimulated by a suitable trigger. A broad spectrum of metallic shape memory alloys has been developed since then [12]. While being able to perform multiple SMEs and generating high forces, these alloys can only generate deformation strains below 8% [13,14]. Nevertheless, several applications, especially in the medical field, require higher strains of at least 100% [15]. Therefore, the development of shape memory polymers (SMPs) represents a promising alternative. SMPs are highly deformable materials consisting of a permanent phase and a switching phase [16,17]. This can either be accomplished by using specifically designed block-co-polymers or by blending two polymers with distinct differences of their glass transition and/or melting temperatures, whereas the lower one defines the necessary activation stimulus for SME. While the permanent phase defines the permanent macroscopic shape, the switching phase acts as a molecular switch. After deformation, the switching phase fixates the temporary shape. Upon (thermal) stimulation, the fixation is released and the original shape is recovered. This effect is driven by the entropy elasticity of the permanent phase.

The implementation of SMP in textile processing technologies enables easy-to-manufacture SME structures with anisotropic properties and a large variety and flexibility of shapes and functions. In particular for minimal invasive medical applications, SMP fibers, yarns, and textiles are of increasing interest and offer new possibilities [16,18]. There are several methods of generating SMP fibers, e.g., melt [15] and wet spinning [19], using various material systems. However, isolated investigations regarding the effect of production methods on the SME were not performed. Therefore, in this paper, the influence of the spinning technology on the shape memory properties are analyzed. Melt spinning and solvent wet spinning are applied for spinning a commercially available, biocompatible SMP (Desmopan 2795-A-SMP). Desmopan is a block-co-polymer with poly(1,4-butylene adipate) as its switching phase. This phase exhibits a melting point at approximately 40 °C, acting as the activation temperature of the SME. Desmopan has been utilized for the generation of 4D-printed artificial muscles [20] and was subject to investigations regarding blending for the modification of shape memory properties [21] and enabling triple-SME [22]. However, SMP fibers/yarns of Desmopan have not been thoroughly investigated. Melt and wet spinning regimes for Desmopan are presented in this paper and the influence of the spinning methods on the shape memory effect are analyzed. Therefore, shape fixation, shape recovery, programming, and generated stress of the yarns are characterized.

## 2. Materials and Methods

Desmopan 2795-A SMP was provided by Covestro AG, Leverkusen Germany. N,N-Dimethylformamide (DMF) was purchased from neoLab Migge GmbH, Heidelberg, Germany.

### 2.1. Thermodynamic Analysis

The SME were characterized by performing differential scanning calorimetry (DSC) measurements on the as-spun Desmopan yarns. The samples were heated to 80 °C (first heating cycle) and then cooled to −20 °C (cooling cycle), then reheated to 80 °C (second heating cycle). The heating rate was set to 2 K/min. Phase transitions were evaluated using the first cooling cycle and the second heating cycle. The heating and cooling cycles of this thermal characterization were used to determine the temperatures for activating the SME and for fixation.

### 2.2. Spinning Conditions for SMP Yarns

The thermoplastic polyurethane Desmopan 2795-A SMP was both melt- and wet-spun to compare influences of the spinning techniques on the SME.

Melt spinning of Desmopan was performed on a melt spinning plant by DIENES Apparatebau GmbH, Germany. The spinning package comprised 40 spinnerets of 300 µm diameter, each.

Prior to solvent wet spinning, Desmopan pellets were dissolved in DMF, generating a 20 wt.% solution that was degassed at 60 °C for 24 h. Spinning was performed on a wet spinning plant by Fourné Maschinenbau GmbH, Germany, comprising one coagulation bath, two washing baths, a curing chamber, and a winder.

Using these methods, three Desmopan 2795-A SMP yarns (see Table 1) were realized. They solely differ in the spinning method and effective draw ratio, which are indicated in the name of the case group (e.g., DesmoW2.2 = material: Desmopan 2795-A SMP; spinning method: wet spinning [W]; draw ratio: 2.2). The yarns were characterized regarding their fineness by weighing and morphology via scanning electron microscopy.

### 2.3. Analysis of Shape Memory Effect—Strain Fixation and Recovery

The SME was analyzed by means of thermo-mechanical load/activation cycles, where each cycle *n* was composed of deformation, fixation, and recovery. The cycles were performed on a Zmart.Pro Z100 tensile testing machine equipped with a temperature chamber (ZwickRoell GmbH & Co. KG, Ulm Germany). Prior to the analysis, the yarns were conditioned at 50 °C to eliminate any previously programmed SME. To determine the characteristics of the SME, the yarns were clamped at a length of 30 mm and stretched to a maximum elongation *ε_m_* of 300% at 100 mm/min, in accordance with [15]. Immediately after that, the elongation of the sample was kept constant at a temperature of 20 °C for 1 min to realize the fixation of the elongation. Subsequently, the clamps of the specimen were positioned at 0% strain, thus, the specimens were in a stress-free condition. Finally, the specimens were stimulated in the temperature chamber at 50 °C for 2 min to realize strain recovery. The next cycle was initiated by cooling the specimen to room temperature and stretching it again to 300%. Per case group, five yarns were evaluated. Each yarn was characterized by four testing cycles. To characterize the SME, the initial strain *ε_p_*(*n*) and the fixed strain *ε_f_*(*n*) of the stress–strain curve of each cycle were recorded. The strain fixation *R_f_* (Formula (1)) and strain recovery *R_r_* (Formula (2)) were calculated from *ε_p_*(*n*) and *ε_f_*(*n*).

(1) $$R_{f}\left(n\right)=\frac{\varepsilon_{f}\left(n\right)-\varepsilon_{p}\left(n-1\right)}{\varepsilon_{m}-\varepsilon_{p}\left(n-1\right)}$$

(2) $$R_{r}\left(n\right)=\frac{\varepsilon_{m}-\varepsilon_{p}\left(n\right)}{\varepsilon_{m}-\varepsilon_{p}\left(n-1\right)}$$

*R_f_*—strain fixation;

*R_r_*—strain recovery;

*ε_f_*—fixed strain;

*ε_p_*—initial strain;

*ε_m_*—maximum strain;

*n*—cycle.

### 2.4. Analysis of Shape Memory Effect—Stress Generation

To characterize the generated force of the SMP yarns, five specimen of each case group were tested using the same machine used for analyzing the shape recovery. Prior to the testing, a conditioning step at 50 °C was performed. The yarns were clamped at a length of 30 mm. First, the SME was programmed by stretching the yarns to 250% of their initial length at 100 mm/min and room temperature. After holding this deformation for 1 min, the strain was set to values of 75% or 150%, and the yarn was heated to 50 °C, activating the SME. After activation, the generated force was analyzed and compared to the force needed to achieve the initial deformation to define the efficiency of the yarn. The resulting stress was calculated using the polymer density of 1.2 g/cm^3^ (according to SDS provided by manufacturer), the yarn fineness, and force (Formula (3)).

(3) $$\sigma =\frac{F\times \rho }{f}$$

*σ*—stress;

*F*—force;

*f*—fineness;

*ρ*—density.

## 3. Results

### 3.1. Thermodynamic Analysis

**Figure 1:**
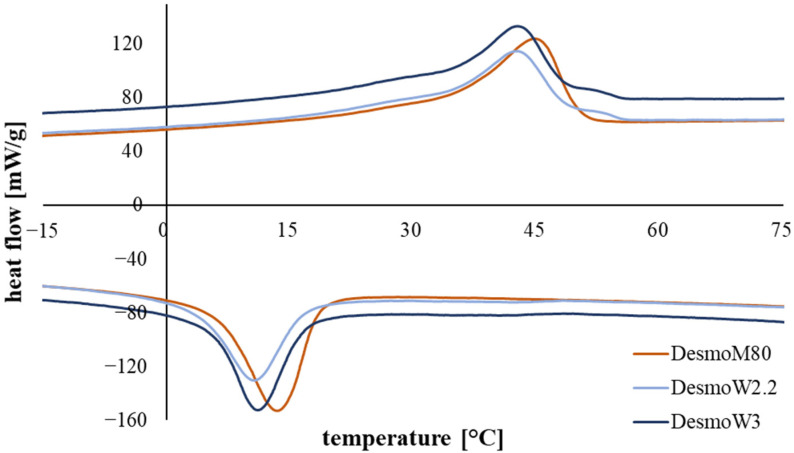
Display of the cooling and 2nd heating cycles of the three Desmopan yarns during DSC; a pronounced phase transformations of the switching phase starts at approx. 40 °C and peaks at 45 °C, respectively. (Re-)Crystallization starts at approx. 20 °C and peaks at 10–15 °C.

DSC measurements show distinct phase transitions of the three Desmopan yarns at 45 °C (endothermal) and 10–15 °C (exothermal) (Figure 1). These correspond to the melting temperature and crystallization temperature of the switching phase of Desmopan, respectively. Desmopan is a block-co-polymer of 1,4-butanediol (permanent phase) and a pre-polymerized polyol of poly(1,4-butylene adipate) (switching phase), which itself displays a SME with a recovery temperature above 40 °C and a fixation temperature below 18 °C. In the temperature range between ~10 °C and ~40 °C, a shoulder is apparent for all case groups during the heating cycle.

### 3.2. Melt and Wet Spinning of SMP Yarns

**Figure 2:**
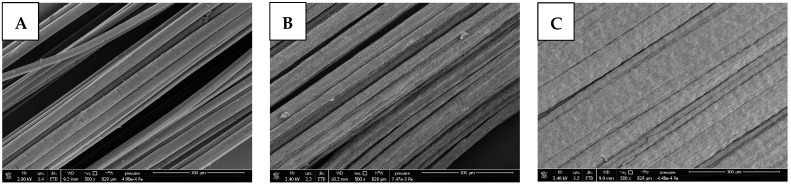
SEM images of the morphologies of melt- and wet-spun SMP yarns: (**A**) DesmoM80, (**B**) DesmoW2.2, and (**C**) DesmoW3. Scale bar = 300 µm.

Both continuous melt and wet spinning of Desmopan were successfully performed, resulting in homogeneous multifilament yarns (Figure 2; only some post-processing artifacts can be seen in Figure 2B). For melt spinning, the SMP was melted and homogenized in an extruder at 200 °C and pressed through the spinnerets at 180–200 bar. The strands were extruded at a speed of ~5.5 m/min and finally wound on a spool at 450 m/min, resulting in a draw ratio of 80. For wet spinning, the polymer was dissolved in DMF and coagulated in water. The spinning package comprised 1008 spinnerets with a diameter of 70 µm. The solution reservoir was kept at 60 °C, the coagulation baths were at room temperature. The as-spun yarns were continuously dried at 50 °C and wound onto a spool at 6.8 m/min and 7.6 m/min. Using six galette duos, drawing ratios of 2.2 and 3 were set. Accordingly, to keep the process stable, the feed pump had to be adjusted from 10 rpm (draw ratio 2.2) to 8 rpm (draw ratio 3), correlating to 9.6 cm^3^/min and 12 cm^3^/min, respectively. Regarding melt spinning, significantly higher production speeds of 450 m/min could be achieved compared to 7.6 m/min with wet spinning. Scanning electron microscopy (SEM) images show that the melt-spun yarns possess a mostly smooth surface with occasional bumps on single filaments (Figure 2A). Surfaces of wet-spun yarns display fibrils on each single filament. With increasing draw ratio this feature is more distinct. Both wet-spun case groups show interlinks of single filaments (Figure 2B,C), resulting in macroscopic band-like structures. Fineness measurements of the three case groups were performed to better characterize the generated SME. An overview of the spinning parameters and yarn characteristics are shown in Table 1.

### 3.3. Analysis of Shape Memory Effect—Strain Fixation/Recovery and Stress Generation

**Figure 3:**
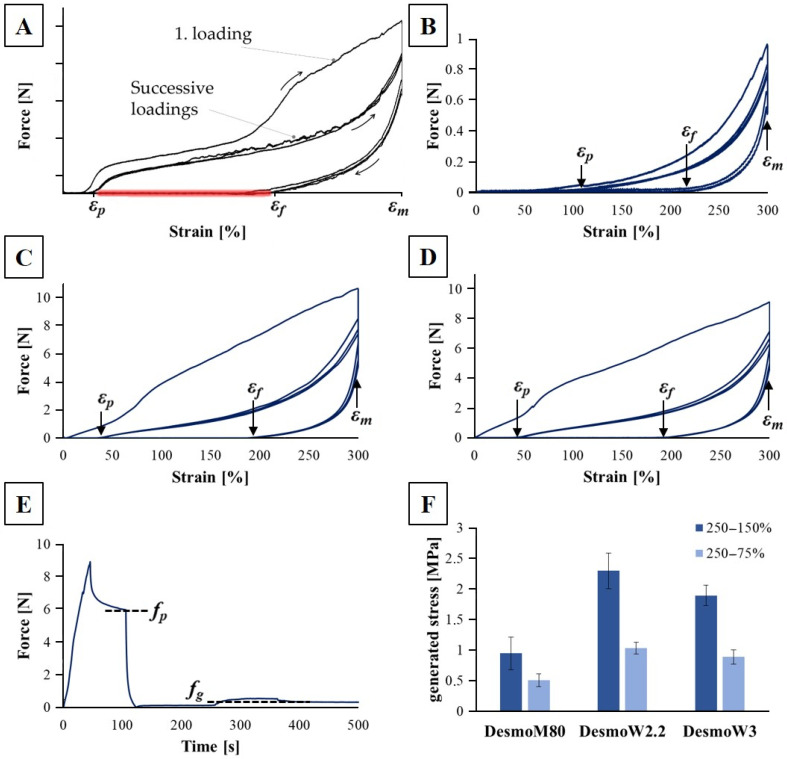
Schematic diagram (**A**) showing the location of relevant strains (initial strain *ε_p_*, fixed strain *ε_f_*, maximum strain *ε_m_*) for calculating strain fixation and recovery. Marked in red is the area of heating/activating the SME. Force–strain curves of SMP yarns during cyclic testing of DesmoM80 (**B**), DesmoW2.2 (**C**), and DesmoW3 (**D**) during cyclic activation at 50 °C. Typical force–time–diagram of SMP yarn (here DesmoW2.2, 250–150%) for determination of programming and generated stress during activation using programming force (*f_p_*) and generated force (*f_g_*) (**E**). Max. generated stress while activation at different leftover strains after programming is shown (**F**).

Static and cyclic tensile tests were performed to characterize the SME. The results of the strain fixation, strain recovery, and generated stress reveal a significant impact of the spinning method and are summarized in Table 2 and shown in Figure 3.

Figure 3A shows the position of relevant strains for calculating *R_f_* and *R_r_*. Figure 3B–D display the force–strain curves generated during cyclic testing of the SME of Desmopan yarns. According to the DSC results, the SME is activated at 50 °C to display the full potential of the material in relatively short cycling times. Regarding the strain fixation (*R_f_*) and recovery (*R_r_*), the data of the first conditioning cycle are neglected and data presented here display the mean values of cycles 2–4. *R_f_* shows a dependency of the spinning method. For melt-spun yarn DesmoM80, approximately 70% of the applied strain could be fixed at room temperature. In the wet-spun yarns DesmoW2.2 and DesmoW3, this value decreases to 55–60%. The strain recovery *R_r_* during shape memory activation is close to 100% for all investigated case groups and cycles (Table 2). No reduction in this value is determined during four cycles (Figure 3B–D).

Figure 3E displays an exemplary force–time–curve (DesmoW2.2, 250–150%) for determination of the programming (*f_p_*) and generated force (*f_g_*) used for calculating the corresponding stress via (3). When activating the heating (approx. at 250 s in Figure 3E), a vent is turned on for homogeneous heat distribution, resulting in an initial higher force detection. *F_g_* is measured after turning the vent off. In stress generation, a clear influence of the spinning method can be seen, regardless of the strain reduction after programming (Figure 3F). While the melt-spun yarn generates up to 0.95 Mpa while contracting, wet-spun yarns are able to generate more than double this amount to 2.29 MPa or 1.89 MPa (depending on the draw ratio). To characterize the generated strain, the yarns had to be brought to a stress-free state after programming. Therefore, the strain is reduced from 250% (programming) to 150% and 75%, resulting in 60% and 30% of the initial strain, respectively. These conditions result in a bisection of the generated stress when reducing the strain from 60% to 30% for all case groups (Table 2 and Figure 3F).

## 4. Discussion

DSC measurements are comparable for all Desmopan yarns, showing no notable differences. This is expected, as all yarns exhibit the same material composition. Distinct phase transitions are apparent for all melt- and wet-spun case groups at approx. 45 °C (offset at ~40 °C) and 10–15 °C (offset at ~20 °C), corresponding with the melting temperature and crystallization temperature of the switching phase of Desmopan, respectively (Figure 1). These values are in good agreement with the literature [21,22]. The determination of the activation and programming temperatures for the SMP yarns during characterization of the SME was based on these results and were set at 50 °C and room temperature, respectively. The temperature was set above the detected peak to fully activate the SME of investigated yarns. Furthermore, a shoulder is apparent during the heating cycles of all three yarns starting at approx. 10 °C and transitioning into the melting peak at 40 °C. Shirole et al. correlate this behavior to nucleating effects of the poly(1,4-butylene adipate) (switching phase) in the SMP [21]. Therefore, it is assumed that the endothermic shoulder corresponds to the melting of smaller crystallites. Nevertheless, investigations show that the SME cannot be triggered at these low temperatures.

The detected fibril surface morphology of wet-spun fibers (Figure 2B,C) can be attributed to the diffusion behavior of solvent during the spinning process. The rapid coagulation in water generates a solid skin that can act as a diffusion barrier, trapping solvent in the fiber core [23,24,25]. During drying, the solvent evaporates eventually, leaving the skin to collapse and form the fibril structure, which is not visible in melt-spun fibers that display a very rapid solidification [26]. This fibril structure becomes more distinct for higher draw ratios, which can be attributed to increased molecular orientation after drawing [27]. Furthermore, the filament interlinkage becomes more distinct for higher draw ratios, too. This can especially be seen in DesmoW3 (Figure 2C), where nearly no distinct filament-like structures can be seen. This can also be attributed to left over solvents in the filament core after initial coagulation of the shell. During the spinning process, diffusion mechanism force this solvent to exit the core. Higher draw ratios enhance the surface–volume ratio of the filaments, increasing the diffusion speed and enhancing the surface to interact with the solvent, resulting in filament interlinkage and, finally, band-like structures.

The SME of the melt- and wet-spun was characterized by analysis of the strain fixation and recovery properties, as well as the generated stresses. All case groups exhibit excellent strain recovery of 100% after the first conditioning cycle. For strain fixation, DesmoM80 achieves 68.76% and, therefore, higher values (+10%) compared to the wet-spun case groups. This correlates with a decreased elastic deformation fraction in melt-spun yarn that can be attributed to the higher draw ratio and, therefore, increased molecular orientation. This is confirmed in comparing wet-spun case groups where an increase in draw ratio results in enhanced strain fixation.

Regarding stress, differences in the programming stress and generated stress are apparent throughout the melt- and wet-spun case groups. According to Formula (3), DesmoM80 exhibits a programming stress (250–150%) of 13.07 MPa vs. 0.95 MPa max. generated stress, resulting in an effective conversion of 7.27%. DesmoW2.2/DesmoW3 achieve values of 39.00/28.84 MPa, 2.29/1.89 MPa, and 5.87/6.55%, respectively. These values are comparable to values in the literature of SMP fibers, which range between 1–3 MPa [28,29]. The programming stress for wet-spun yarns is more than doubled, owing to the high surface roughness and filament interlinks. Furthermore, wet-spun fibers display high molecular orientation in their skin area [30,31,32,33], adding to higher crystallinity and, therefore, increased programming stress. This is attributed to shear-induced orientation in the spinning nozzle and very fast coagulation in supersaturated systems, as present in the employed wet spinning regime [32,34]. The core areas are characterized by less crystallinity and molecular orientation that can positively influence the drawing behavior, enabling higher plastic deformation during programming and, therefore, enlarging the generated stress during activation of the SME. This can also explain the decrease in generated stress for higher draw ratios (DesmoW3) due to increased molecular orientation in the unprogrammed state. Melt-spun fibers, on the other hand, are characterized by a fast and homogeneous crystallization throughout the fiber cross-section [26]. The effective conversion of programming to generated stress is comparable throughout all case groups, with a slight increase for melt-spun fibers.

Furthermore, the generated stress shows a clear dependency on the pre-strain condition of the programmed SMP. When reducing the programming strain to a value of 60%, the generated stress is doubled compared to 30%. When the SME is activated, stress can only be measured when the yarn is clamped in tension. Therefore, the starting point of the measurement should be matched to the strain fixation of the specimens in order to measure the maximum generated stress, which is the case at 60%. At lower initial strains, the SME initially only contributes to the tightening of the yarn before a stress can be measured. Thus, the doubling of the generated stress shows that a continuous increase in stress can be expected as the SMP continues to contract.

## 5. Conclusions

SMP yarns were generated using melt and wet spinning. Both manufacturing methods were compared regarding the characteristics of the SME. Furthermore, influences of the draw ratio on the SME were characterized. Using wet spinning, higher activations stresses of up to 2.3 MPa could be achieved, while stress conversion is comparable to melt-spun yarns. Melt-spun yarns are superior concerning strain fixation, which is attributed to increased molecular orientation. They are able to conserve 70% of the applied programming strain. Regarding strain recovery, all case groups exhibit excellent behavior of 100% recovery for the analyzed testing cycles.

## Figures and Tables

**Table 1:** Overview of spinning parameters and yarn characteristics of the three case groups.

Case Group	Spinning Method	Fineness (tex = g/km)	Drawing Ratio	Drawing Speed (m/min)	Feed Rate (cm^3^/min)
DesmoM80	Melt spinning	42	80	450	15.75
DesmoW2.2	Wet spinning	268	2.2	6.8	9.6
DesmoW3	Wet spinning	180	3	7.6	12

**Table 2:** Characteristics of SME of analyzed Desmopan–PCL–bend yarns.

Case Group	Strain Fixation *R_f_* (%)	Strain Recovery *R_r_* (%)	Max. Generated Stress 250–150% (MPa)	Max. Generated Stress, 250–75% (MPa)
DesmoM80	68.76 ± 4.00	100.44 ± 7.05	0.95 ± 0.27	0.51 ± 0.10
DesmoW2.2	54.89 ± 2.20	100.08 ± 0.38	2.29 ± 0.29	1.03 ± 0.09
DesmoW3	58.20 ± 2.08	100.39 ± 1.27	1.89 ± 0.16	0.89 ± 0.12

## Data Availability

The data presented in this study are available on request from the corresponding author.
